# From seeds to survival rates: investigating *Linum usitatissimum*’s potential against ovarian cancer through network pharmacology

**DOI:** 10.3389/fphar.2023.1285258

**Published:** 2023-10-30

**Authors:** Mohammed Monirul Islam, Nagaraja Sreeharsha, Fahad M. Alshabrmi, Afzal Haq Asif, Bandar Aldhubiab, Md Khalid Anwer, Rajendiran Krishnasamy, Abdur Rehman

**Affiliations:** ^1^ Department of Biomedical Sciences, College of Clinical Pharmacy, King Faisal University, Al-Ahsa, Saudi Arabia; ^2^ Department of Pharmaceutical Sciences, College of Clinical Pharmacy, King Faisal University, Al-Ahsa, Saudi Arabia; ^3^ Department of Pharmaceutics, Vidya Siri College of Pharmacy, Bangalore, India; ^4^ Department of Medical Laboratories, College of Applied Medical Sciences, Qassim University, Buraydah, Saudi Arabia; ^5^ Department of Pharmacy Practice, College of Clinical Pharmacy, King Faisal University, Al-Ahsa, Saudi Arabia; ^6^ Department of Pharmaceutics, College of Pharmacy, Prince Sattam Bin Abdulaziz University, Al-Kharj, Saudi Arabia; ^7^ Department of Pharmacognosy, Vidya Siri College of Pharmacy, Bangalore, India; ^8^ Center of Bioinformatics, College of Life Sciences, Northwest A&F University, Yangling, China

**Keywords:** ovarian cancer, *Linum usitatissimum*’s, network pharmacology, molecular docking, molecular dynamic simulation

## Abstract

Ovarian cancer is a malignant tumor that primarily forms in the ovaries. It often goes undetected until it has spread to the pelvis and abdomen, making it more challenging to treat and often fatal. Historically, natural products and their structural analogues have played a pivotal role in pharmacotherapy, especially for cancer. Numerous studies have demonstrated the therapeutic potential of *Linum usitatissimum* against ovarian cancer, but the specific molecular mechanisms remain elusive. This study combines data mining, network pharmacology, and molecular docking analysis to pioneer an innovative approach for ovarian cancer treatment by identifying potent phytochemicals. Findings of current study revealed that Apigenin, Vitamin E, Palmitic acid, Riboflavin, Isolariciresinol, 5-Dehydro-avenasterol, Cholesterol, Pantothenic acid, Nicotinic acid, Campesterol, Beta-Sitosterol, Stigmasterol, Daucosterol, and Vitexin suppress tumor growth by influencing AKT1, JUN, EGFR, and VEGFA. Kaplan–Meier survival analysis spotlighted AKT1, JUN, EGFR, and VEGFA as potential diagnostic and prognostic biomarkers for ovarian cancer. However, it is imperative to conduct *in vivo* and *in vitro* examinations to ascertain the pharmacokinetics and biosafety profiles, bolstering the candidacy of *L. usitatissimum* in ovarian cancer therapeutics.

## 1 Introduction

Ovarian cancer, originating from the ovaries, is one of the most deadly gynecological malignancies affecting women worldwide (Bray et al., 2018). Globally, it stands as the seventh most common cancer in women and is often termed the “silent killer” due to its insidious onset and late-stage diagnosis (Reid et al., 2017). Such late diagnosis is linked to its non-specific symptoms, like abdominal discomfort, bloating, and urinary symptoms, which can easily be misattributed to less severe conditions (Goff et al., 2000). Histologically, ovarian cancers can be classified into several types, with epithelial ovarian cancers (EOC) being the predominant form, accounting for approximately 90% of cases (Kurman and Shih, 2010). These are further subdivided into serous, endometrioid, mucinous, and clear cell carcinomas, among others, each with its unique molecular signature and clinical course (Prat, 2012).

Several risk factors have been identified that increase the likelihood of developing ovarian cancer. These include older age, inherited gene mutations such as BRCA1 and BRCA2, a family history of ovarian or breast cancer, and conditions like endometriosis (Walsh and King, 2007). However, certain factors, including oral contraceptive use, multiple pregnancies, and breastfeeding, have been shown to reduce risk (Cibula et al., 2011). The landscape of ovarian cancer treatment is ever-evolving, with surgical removal of the tumor remaining as the cornerstone of management. This is often followed or complemented by chemotherapy regimens, primarily comprising platinum-based drugs. In recent years, targeted therapies, especially PARP inhibitors, have been introduced, offering hope to patients, especially those with BRCA mutations (Mirza et al., 2016).

*Linum usitatissimum*, commonly known as flaxseed, has gained considerable attention for its potential health benefits, particularly in the context of cancer prevention and treatment (Morris, 2007). The seeds are a rich source of essential fatty acids, lignans, and dietary fiber, all of which have been shown to possess anti-carcinogenic properties (Adolphe et al., 2010). Recent research has begun to explore the role of flaxseed and its constituents in ovarian cancer. Recent studies, such as research conducted by Viveky et al. (Viveky et al., 2015) reported that secoisolariciresinol diglycoside (SDG), a lignan abundant in flaxseed, exhibited anti-proliferative effects on ovarian cancer cells. The lignans in flaxseed have also been shown to act as phytoestrogens, which can modulate estrogen metabolism and signaling pathways associated with cancer development (Flower et al., 2014). Moreover, animal studies have shown that the incorporation of flaxseed in the diet could lead to a reduction in tumor growth rate in ovarian cancer models (Saggar et al., 2010). This effect is potentially attributed to the alpha-linolenic acid (ALA), an omega-3 fatty acid abundant in flaxseed, which has demonstrated anti-inflammatory and anti-carcinogenic properties (Fritsche, 2015). Although these preliminary studies are promising, more extensive research, particularly in clinical settings, is required to establish the efficacy of flaxseed and its bioactive compounds in the treatment or prevention of ovarian cancer. This opens a novel avenue for researchers interested in natural alternatives or adjuncts to current therapeutic strategies for ovarian cancer (Vellingiri et al., 2020).

In recent years, network pharmacology has gained prominence as a method for uncovering the therapeutic potential of medicinal plants, particularly in the context of complex diseases such as ovarian cancer (Noor et al., 2022; Basavarajappa et al., 2023; Noor et al., 2023). This approach takes a holistic perspective, examining how herbal medicine impacts molecular interactions within the body. Previous studies have demonstrated the effectiveness of network pharmacology in predicting the bioactive compounds found in herbal remedies (N et al., 2022; Qasim et al., 2023; Rehman et al., 2023). In this study, we use network pharmacology to identify the bioactive components of *L. usitatissimum* and explore their potential effectiveness against ovarian cancer. Our primary objective is to use network pharmacology to identify the bioactive compounds within *L. usitatissimum* that may exhibit therapeutic properties against ovarian cancer. We aim to understand the mechanisms through which these compounds work and how they interact with specific target proteins. To validate our findings, we conduct molecular docking studies, which confirm the potential interactions between the identified bioactive compounds and their target proteins. Furthermore, we use molecular dynamic (MD) simulations, running for 100 nanoseconds, to investigate the stability and dynamics of these interactions. Importantly, this study represents a pioneering effort in exploring the potential of *L. usitatissimum* in the context of ovarian cancer. The insights gained from this research not only shed light on the therapeutic potential of *L. usitatissimum* but also provide a foundation for future experimental studies in this field.

## 2 Materials and methods

### 2.1 Screening of active compounds

The compound collection was synthesized from data aggregated from esteemed sources, namely, TCMSP (version 2.3) (Ru et al., 2014), IMPPAT (Mohanraj et al., 2018), and KnapSack (Nakamura et al., 2013). Post-aggregation, an exhaustive absorption, distribution, metabolism, and excretion (ADME) screening was employed to discern compounds exhibiting optimal pharmacokinetic characteristics while excluding those with subpar drug-associated attributes. Compounds advancing in the evaluation had to adhere to two salient benchmarks: achieve an Oral Bioavailability (OB) in excess of 30% and register a Drug Likeness (DL) coefficient of greater than 0.18. OB delineates the fraction of an orally-administered compound capable of infiltrating the bloodstream, thereby executing its intended therapeutic role (Batool et al., 2022a). By adhering to an OB baseline of 30%, this methodology aligns with established norms in pharmaceutical research, acknowledging that compounds with values beneath this threshold potentially grapple with compromised efficacy due to inhibited absorption. Conversely, the DL coefficient furnishes a qualitative assessment of a compound’s potentiality as a proficient oral drug. The criteria OB > 0.30 and DL > 0.18 are indicative of a rigorous screening process for compounds with the potential to become effective orally administered drugs. An OB value greater than 0.30 signifies that these compounds have a high likelihood of being well-absorbed into the bloodstream when taken orally, suggesting their practicality for oral delivery (Batool et al., 2022b). On the other hand, a DL score surpassing 0.18 reflects the compound’s similarity to established drugs, which is a crucial aspect of drug development (Asghar et al., 2023). It suggests that these compounds exhibit key characteristics that align with known pharmaceuticals, making them promising candidates for further investigation and development in the realm of drug discovery. These criteria collectively help identify compounds with the desired attributes for oral drug delivery, offering a more efficient path toward potentially impactful pharmaceutical solutions. Employing software tools such as Molsoft and SwissADME, compounds were selected based on their surpassing of OB and DL thresholds set at 30% and 0.18, respectively.

For further refinement, molecular weight parameters and 2D structural configurations of the predicted compounds were sourced from authoritative repositories, notably PubChem and Molinspiration (Kolarevic et al., 2014; Kim et al., 2021). In order to ascertain genes symbiotically linked to these prioritized compounds, platforms like STITCH [(Gfeller et al., 2014) and Swiss Target Prediction (Kuhn et al., 2007) were utilized, with *Homo sapiens* designated as the primary taxonomic reference. Consistency in gene nomenclature and taxonomic designations was maintained through reliance on the UniProt KB database, minimizing ambiguities related to protein variant classifications or postulated pseudogene derivations.

### 2.2 Elucidation of ovarian cancer-associated targets

For the identification of molecular targets intricately associated with ovarian cancer, a bifurcated strategy was employed, emphasizing specific databases known for their comprehensive genetic datasets. Firstly, the GeneCards database, a renowned genomic compendium, was consulted (Safran et al., 2010). This platform, replete with detailed data on human genetic markers, facilitated the delineation of potential genes exhibiting a robust correlation with ovarian cancer phenotypes. After that, the OMIM database, an public repository comprising over 15,500 gene-centric entries, was employed to further consolidate the understanding of genotype-phenotype relationships, particularly in the context of ovarian cancer (Hamosh et al., 2000). The analytical lens was exclusively trained on proteins typifying *H. sapiens*. During this evaluative process, meticulous scrutiny was afforded to details such as the nature of protein-ligand interactions, the methodologies employed for crystal structure elucidation, and the accompanying resolution metrics.

### 2.3 Construction of the PPI network

To elucidate the intricate protein-protein interactions (PPI) pertinent to ovarian cancer and the active constituents of the botanical mixture, the STRING database (https://string-db.org/, ver. 11.0) was consulted [29]. The exploration was confined to proteins representative of *H. sapiens*. A stringent confidence threshold of ≥0.700 was instituted to ensure the derivation of relevant and meaningful associations, subsequently facilitating the generation of a comprehensive PPI network. This methodology was instrumental in highlighting putative interaction dynamics between the botanical mixture’s active components and ovarian cancer-linked proteins.

### 2.4 Analysis via the KEGG pathway

A deep dive into the biological intricacies steered by the pinpointed targets was executed via the Kyoto Encyclopedia of Genes and Genomes (KEGG) pathway enrichment analysis, leveraging the clusterProfiler package in R [30]. This analytical step not only substantiates the integrated findings’ credibility but also illuminates critical biological conduits pivotal in ovarian cancer’s manifestation. KEGG terms that met the stringent criteria of a *p*-value ≤0.05 were earmarked for an in-depth perusal and subsequent interpretation. This pathway-centric exploration offers insights into the conceivable mechanisms through which the botanical blend’s constituents could mitigate ovarian cancer manifestations.

### 2.5 Network analysis

Using the Cytoscape software platform (ver. 3.5.0) (Shannon et al., 2003), an in-depth network analysis was conducted to decipher the intricate relationships between compounds, their specific target interactions, and their affiliations with proteins implicated in ovarian cancer. Specifically, three key networks were constructed: compound-target interactions, compound-ovarian cancer protein relationships, and a comprehensive map detailing the interactions of the botanical blend with ovarian cancer-associated proteins. Through the evaluation of crucial network metrics, notably “Degree”, we identified central proteins, which are referred to as hub genes. These hub genes indicated significant network interconnectivity and are posited to play a fundamental role in the context of ovarian cancer.

### 2.6 Prognostic evaluation

For the conclusive hub genes, prognostic evaluation was executed using the GEPIA2 platform (http://gepia2.cancer-pku.cn/) (Tang et al., 2019). Survival estimations based on gene transcription levels can shed light on the clinical relevance of specific genes. Within the GEPIA2 interface, Kaplan-Meier survival plots were derived from the Ovarian Carcinoma dataset to probe the correlation between hub genes and patient survival outcomes in ovarian malignancies. The gene normalization tool in GEPIA facilitates the comparative transcription analysis of two separate genes provided as input. Hub genes that manifested a *p*-value less than 0.05 were deemed statistically relevant and subsequently directed to molecular docking assessments.

### 2.7 Validation of multi-target effect of active compounds using molecular docking

For the verification of interactions between compounds and their corresponding targets, molecular docking simulations were performed using Autodock Vina 1.1.2 within the PyRx 0.8 framework (Dallakyan and Olson, 2015). The underlying algorithm of PyRx AutoDock Vina is recognized for its precise and efficient docking predictions. The RCSB Protein Data Bank (PDB) database (http://www.rcsb.org/) (Kouranov et al., 2006) served as the source for obtaining 3D configurations of the target proteins. During the pre-docking phase, extraneous water molecules were eliminated, protein configurations were refined, and only those regions pivotal to binding were retained. In parallel, the active botanical compounds were optimized to achieve ideal geometries for docking scenarios. Once prepared, these ligands and the refined proteins underwent docking via the PyRx AutoDock Vina interface. This step entailed positioning the ligands within the protein’s binding pocket and subsequently evaluating binding energies. The primary objective of utilizing PyRx AutoDock Vina was to corroborate and delve deeper into the associations between the botanical compounds and their target proteins. Through these simulations, detailed understanding of potential binding patterns and strengths was attained, reinforcing the legitimacy of the identified compound-protein interactions. The acquired results bolster the comprehension of molecular associations instrumental to the medicinal properties of the botanical blend in relation to ovarian cancer.

### 2.8 Molecular dynamic (MD) simulation

Molecular dynamics (MD) simulation serves as a computational approach that meticulously tracks and delineates atom interactions and trajectories within a specified system (Abbas et al., 2022; Shams ul Hassan et al., 2022; Aqeel et al., 2023). With an emphasis on each individual atom, MD approaches solve motion equations while considering the interatomic forces that dictate atomic interactions. For the current study, GROMACS 2018 was the chosen tool for executing MD simulations (Khan et al., 2022a; Khan et al., 2022b). The DockPrep utility was leveraged to refine the system’s structural configuration prior to MD simulations (Pettersen et al., 2004). The protein’s structural framework was delineated using the OPLS-AA/L force field, whereas the ligands received their parameters from the SwissParam platform (Zoete et al., 2011). Adhering to established methodologies from earlier studies (Rehman et al., 2022), MD analyses spanned a timeline of 20 nanoseconds (ns). To extract insights about the simulation’s nuances, we evaluated metrics such as the root mean square fluctuation (RMSF), the system’s radius of gyration, and the root mean square deviation (RMSD). Such metrics illuminate the system’s dynamism, structural stability, and potential conformational shifts throughout the simulation.

### 2.9 Binding affinity calculation

Binding free energy estimations play a pivotal role in molecular dynamics simulations by offering insights into the stability of protein-ligand interactions. Such estimations shed light on the binding affinity between proteins and ligands, revealing shifts in thermodynamics when these molecules bind [41]. This knowledge assists in forecasting the propensity of specific protein-ligand complex formation and its robustness under various environments. Notably, MMGBA/PBSA methods, which amalgamate molecular mechanics energies with either Poisson–Boltzmann or generalized Born and surface area solvation models, emerge as a precise alternative to the conventional scoring techniques prevalent in protein-ligand docking studies. In our research, we employed the MMGBA/PBSA method to deduce the binding free energies (DG) by averaging over 1,000 simulation frames. The net DG was discerned by contrasting the DG values of the individual ligand, protein, and their collective complex as depicted in Eq. 2.1:

$$∆G_{\text{bind}}=∆G_{\text{complex}}-∆G_{\text{receptor}}-∆G_{\text{ligand}}$$ (2.1)

The ensuing DG epitomizes the Gibb’s free energy, evaluated through MMGB/PBSA, illustrated in Eq. 2.2:

$$∆G=∆E_{\text{gas}}+∆G_{\text{solv}}-∆T_{\text{Ssolute}}$$ (2.2)

Here $E_{\text{gas}}$ signifies molecular mechanics force field-derived energy, while “T” and “S” delineate the temperature and entropy affiliated with ligand binding, in that order. The $E_{\text{gas}}$ component comprises electrostatic energies, intrinsic energy, and interactions akin to van der Waals forces. The solvation component $\left(∆G_{\text{solv}}\right.$ ), hinged on the implicit solvent model, is a blend of polar solvation and electrostatic energy as defined in Eqs 2.3 and 2.4:

$$∆G_{\text{solv}}=∆G_{\text{ele}}+∆G_{\text{np}}$$ (2.3)

$$∆G_{\text{ele}}\text{PB}/\text{GB}=E_{\text{ele}}+{∆}_{\text{GPB}/\text{GB}}$$ (2.4)

Within Eq. 2.3, $∆G_{\text{np}}$ corresponds to the non-electrostatic component, tied to the solvent-exposed surface area of the molecule. This metric is deduced using the Linear Combinations of Pairwise Overlaps technique in MMPBSA [41]. In the context of Eq. 2.4, “GB” and “PB” denote generalized born and Poisson-Boltzmann models, respectively.

$$∆G_{\text{np}}=\gamma \text{SAS}+\beta$$

In Equation (2.5), the standard values used were 0.0072 kcal/mol.Å2 and 0 kcal/mol for the γ and *ß* constants, respectively, in MMGBSA, and 0.0052 kcal/mol.Å2 and 0.92 kcal/mol in MMPBSA.

## 3 Results

### 3.1 Extraction of putative bioactive constituents

Following a rigorous bioinformatics analysis, nine phytochemical constituents were delineated from *L. usitatissimum*. Upon eliminating redundant entries, the dataset was consolidated to a total of 14 distinct phytochemicals including Apigenin, Vitamin E, Palmitic acid, Riboflavin, Isolariciresinol, 5-Dehydro-avenasterol, Cholesterol, Pantothenic acid, Nicotinic acid, Campesterol, Beta-Sitosterol, Stigmasterol, Daucosterol, and Vitexin, all of which are belonging to *L. usitatissimum*. These compounds adhered to the specified pharmacokinetic criteria: each exhibited a Drug Likeness (DL) coefficient of ≥0.18, demonstrated an Oral Bioavailability (OB) parameter of ≥0.30, and possessed a molecular mass under the 500 g/mol threshold. Their congruence with these stringent benchmarks positions them as formidable contenders for subsequent pharmacodynamic evaluations. A comprehensive profile, encompassing structural motifs, physicochemical properties, and tentative therapeutic applications of these compounds, is systematically delineated in Table 1.

**TABLE 1:** Phytochemical characteristics of active constituents.

Phytochemicals	Oral bioavailability (OB)	Drug likeness (DL)	2D structure
Apigenin	0.55	0.39	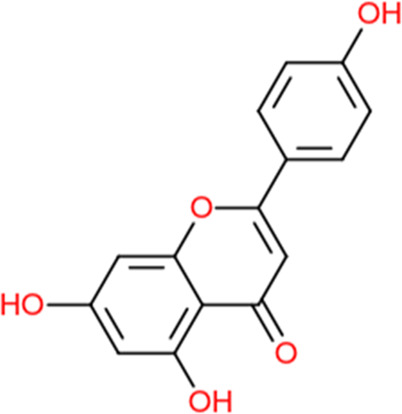
Vitamin E	0.55	0.48	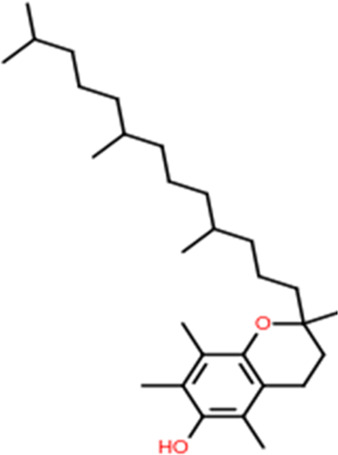
Palmitic acid	0.85	0.54	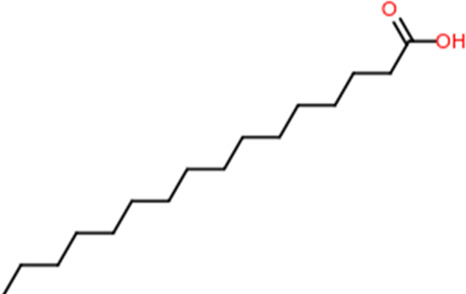
Riboflavin	0.55	0.62	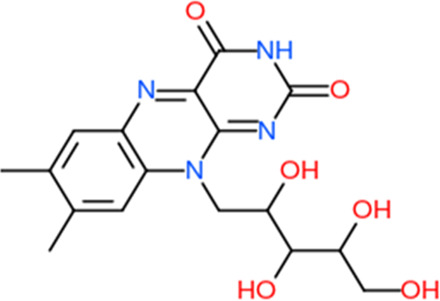
Isolariciresinol	0.55	0.96	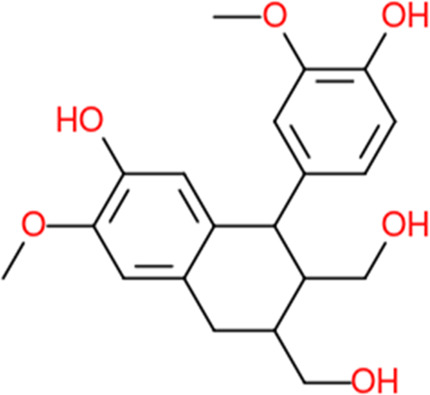
5-Dehydro-avenasterol	0.55	0.57	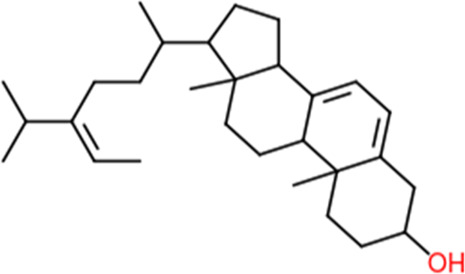
Cholesterol	0.55	0.49	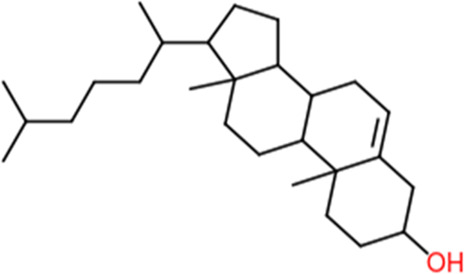
Pantothenic acid	0.56	0.62	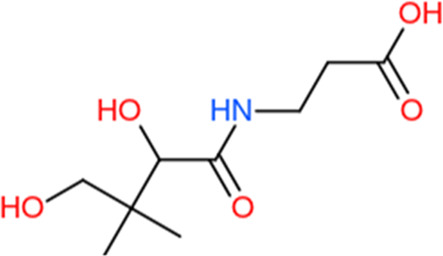
Nicotinic acid	0.85	0.3	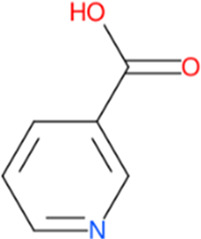
Campesterol	0.55	0.59	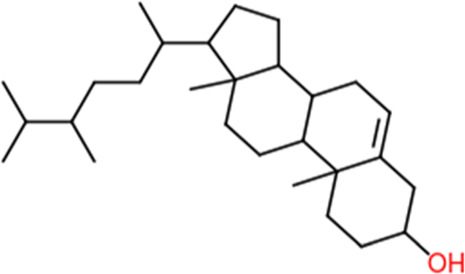
beta-Sitosterol	0.55	0.78	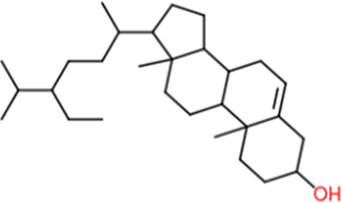
Stigmasterol	0.55	0.62	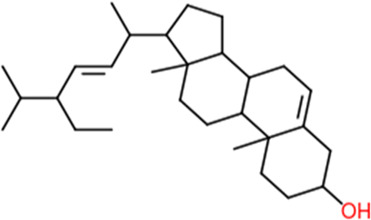
Daucosterol	0.55	0.5	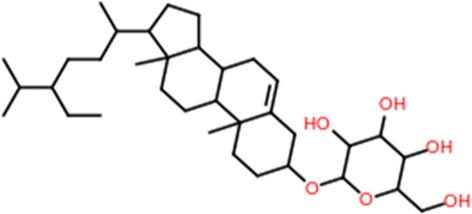
Vitexin	0.55	0.6	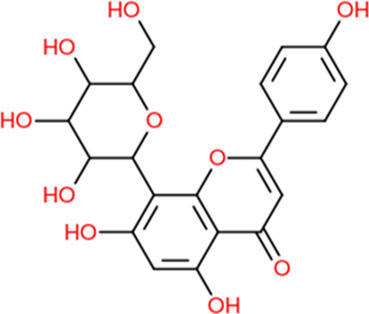

### 3.2 Evaluation of interactions between compounds and targets

**FIGURE 1:**
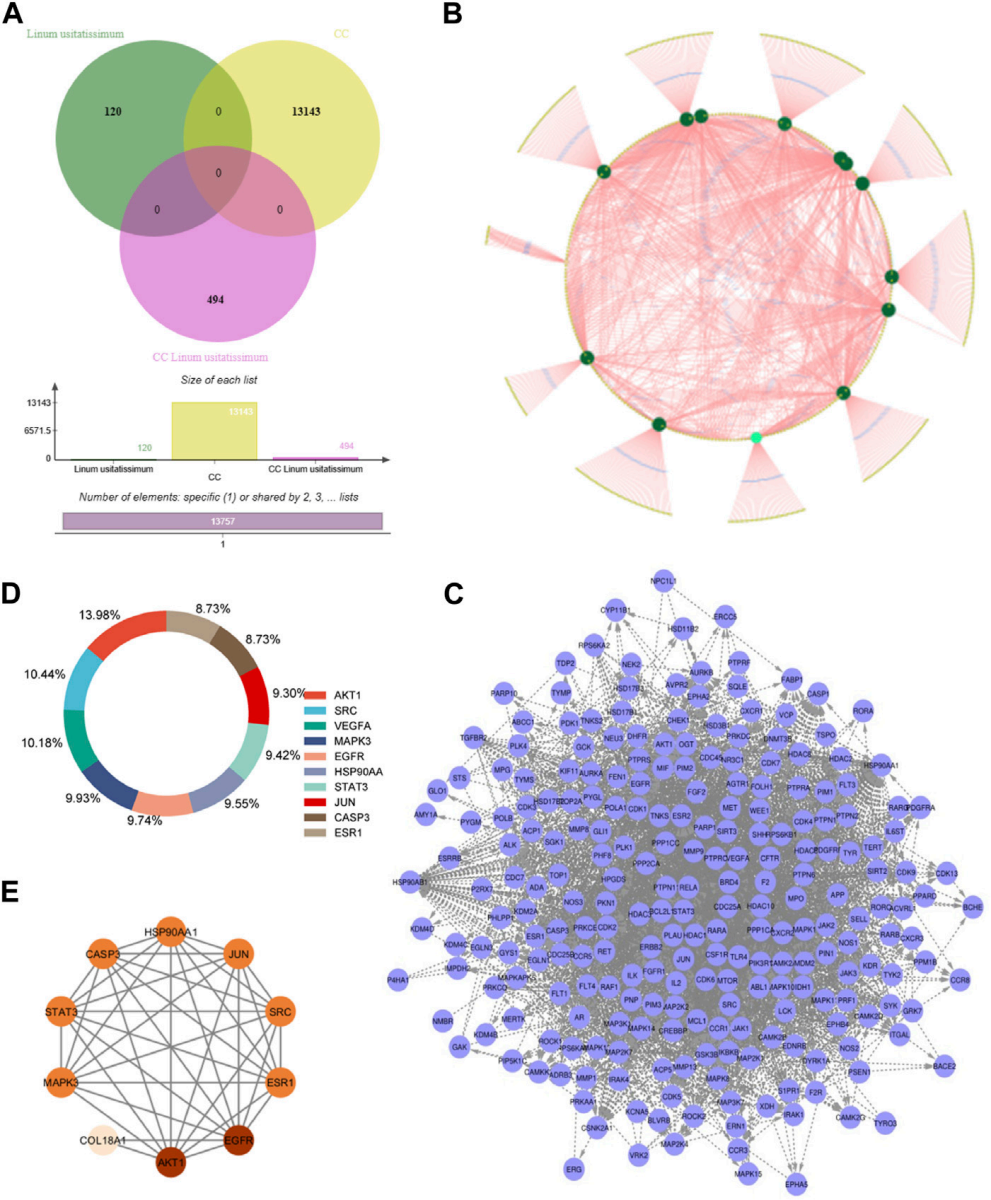
Integrated Analysis of Genes and Compound-Target Networks: **(A)** A Venn diagram illustrating the intersection of 494 genes common to both ovarian cancer and the studied plants. This overlap suggests potential therapeutic or mechanistic intersections between the phytochemicals in the plants and the pathological processes in ovarian cancer. **(B)** Compound-Target Network: This visually represents the interaction between the identified phytochemicals and their associated genetic targets. Nodes within the network vary in color and size, indicating their relative degree of connectivity. Larger and more vividly colored nodes have higher connectivity, signifying their central role within the network. **(C)** Protein-Protein Interaction (PPI) Network for the 494 genes: This graphical representation underscores the complex interplay among the identified genes, depicting their mutual interactions and offering insights into potential pathways or mechanisms that could be harnessed or disrupted for therapeutic advantage. **(D)** Identification of Hub Genes: From the intricate PPI network, the top 10 genes, recognized as “hub genes,” were distilled based on their extensive degree of connectivity. **(E)** PPI Network of Hub Genes: A narrowed-down interaction analysis focusing exclusively on the identified hub genes, emphasizing their mutual interactions and suggesting a core sub-network that might be especially critical in the context of ovarian cancer and the plants under study.

In our investigative analysis, we profiled the compounds and deduced a set of 610 prospective targets (Figure 1A). The compounds under consideration comprised Apigenin, Vitamin E, Palmitic acid, Riboflavin, Isolariciresinol, 5-Dehydro-avenasterol, Cholesterol, Pantothenic acid, Nicotinic acid, Campesterol, Beta-Sitosterol, Stigmasterol, Daucosterol, and Vitexin. Subsequent stages of the study incorporated these proteins for a more detailed examination. This facilitated the creation of an intricate network delineating the interplay between the aforementioned compounds and their corresponding target proteins (Figure 1B). This interaction network features 422 nodes, out of which 409 represent target nodes and the remaining 14 symbolize compound nodes, and is further augmented by 782 edges. Within this network construct, an observation of significance is the multifaceted interaction of certain compounds with a variety of targets, suggesting the potential of multitarget regulation. The implication drawn from this is that the compounds sourced from *L. usitatissimum* could potentially exert a combined effect on these targets, thereby modulating therapeutic responses in disease conditions.

### 3.3 Network analysis

Through a comprehensive juxtaposition of the identified compound targets and the established 10,710 ovarian cancer-associated targets, we discerned a concurrent subset comprising 496 genes (Figure 1A). This convergence points towards a probable association between the compounds’ biological functionalities and ovarian cancer-driven processes. Subsequently, a PPI network for these 343 genes was derived using the STRING database (Figure 1C). This mapped network, marked by notable interconnectivity (degree ≥0.700), encompasses 494 nodes linked by 1,297 connections. Importantly, within this PPI architecture, specific nodes such as AKT1, SRC, VEGFA, MAPK3, EGFR, HSP90AA1, STAT3, JUN, CASP3, and ESR1 exhibit pronounced connective prominence (Figure 1D). The high count of their interactions with other nodes attests to their centrality: for instance, AKT1 has interactions with 221 proteins, SRC has interactions with 165 proteins, VEGFA has interactions with 161 proteins, MAPK3 had has interactions with 157 proteins, EGFR has interactions with 154 other proteins and so forth. These nodes are suggestive of their influential roles in ovarian cancer-centric pathways and the consequential effects of the compounds under investigation.

### 3.4 GO enrichment analysis

**FIGURE 2:**
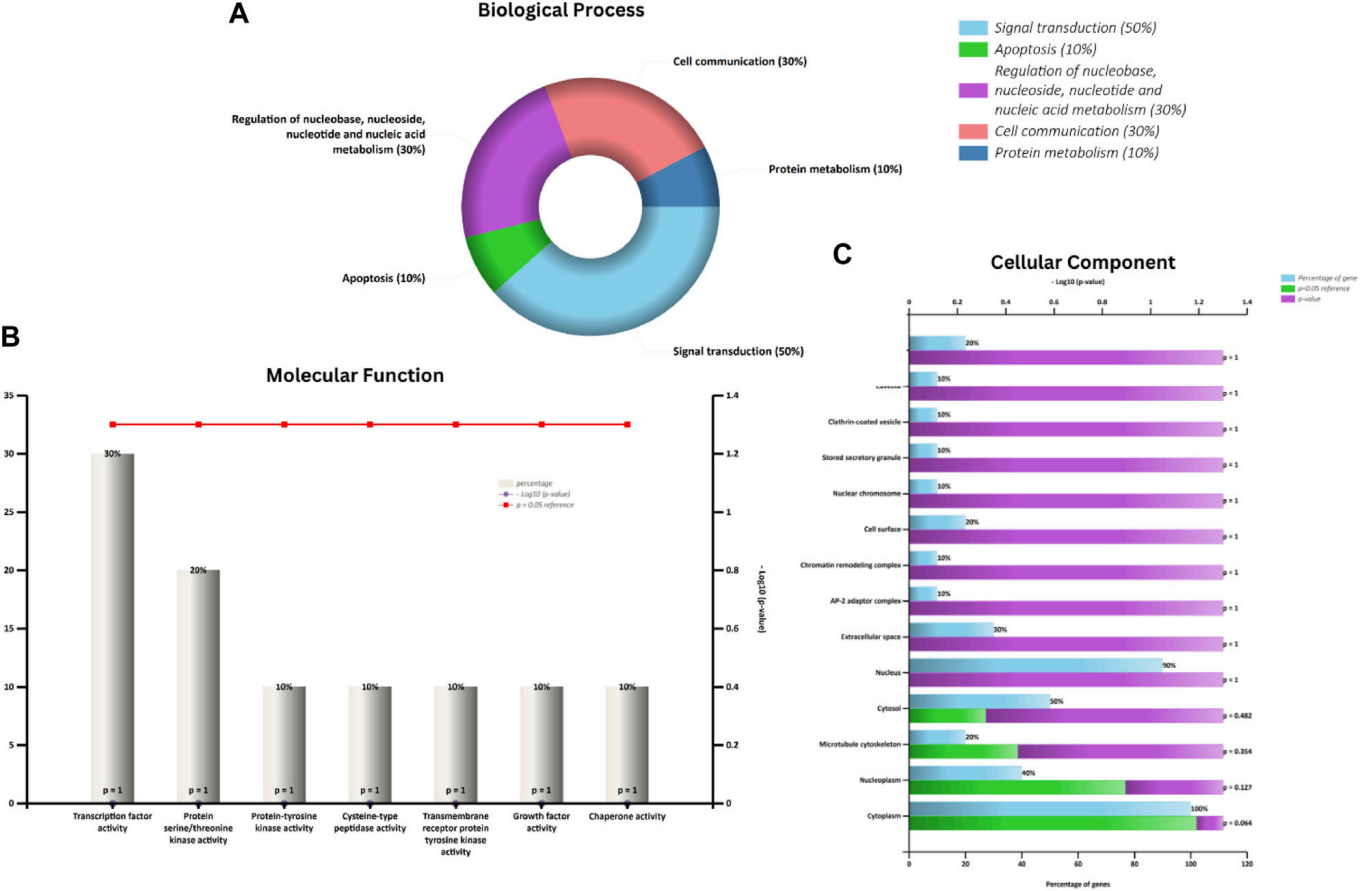
Represents an analysis of Gene Ontology. Panel **(A)** highlights the Biological Process (BP). Panel **(B)** explores the Molecular Function (MF), and **(C)** depicts the Cellular Component (CC).

In the investigation of gene functionalities, multiple significant Gene Ontology (GO) terms associated with biological processes (BP), cellular components, and molecular functions were discerned. The target genes were found to be involved in 93 biological processes, 42 cellular components, and 75 molecular functions. With respect to BP, the genes were notably involved in cellular responses to cadmium ions and reactive oxygen species. Furthermore, these genes played crucial roles in regulating processes such as protein phosphorylation, the ERK1 and ERK2 cascade, and transcription from RNA polymerase II promoter. They were also seen to influence peptidyl-serine phosphorylation, protein kinase B signaling, peptidyl-tyrosine autophosphorylation, and the apoptotic process while also supporting cell proliferation (Figure 2A; Supplementary File S1; Supplementary Table S1). When examining cellular components, our genes localized to a range of cellular sites, including the cytoplasm, nucleoplasm, and plasma membrane (Figure 2B; Supplementary File S1; Supplementary Table S2). Their presence was also evident in macromolecular complexes, the nucleus, and dendritic growth cones. Further, they were associated with structures such as the transcription factor complex, membrane rafts, neuronal cell bodies, glutamatergic synapses, and focal adhesions. From a molecular function standpoint, these genes exhibited a diverse array of roles (Figure 2C; Supplementary File S1; Supplementary Table S3). They participated in nitric-oxide synthase regulation, enzyme binding, protein homodimerization, and protein kinase activity, among others. Their involvement extended to ubiquitin protein ligase binding, chromatin binding, estrogen receptor binding, and transcriptional activator activity, emphasizing their versatility. Additionally, some genes exhibited enzyme inhibitor activity and had affinities for binding to proteins and ATP. Overall, the profound involvement of these genes in a multitude of cellular activities accentuates their potential importance in the biological systems being studied.

### 3.5 In-depth pathway analysis of *L. usitatissimum* compounds in ovarian cancer

**FIGURE 3:**
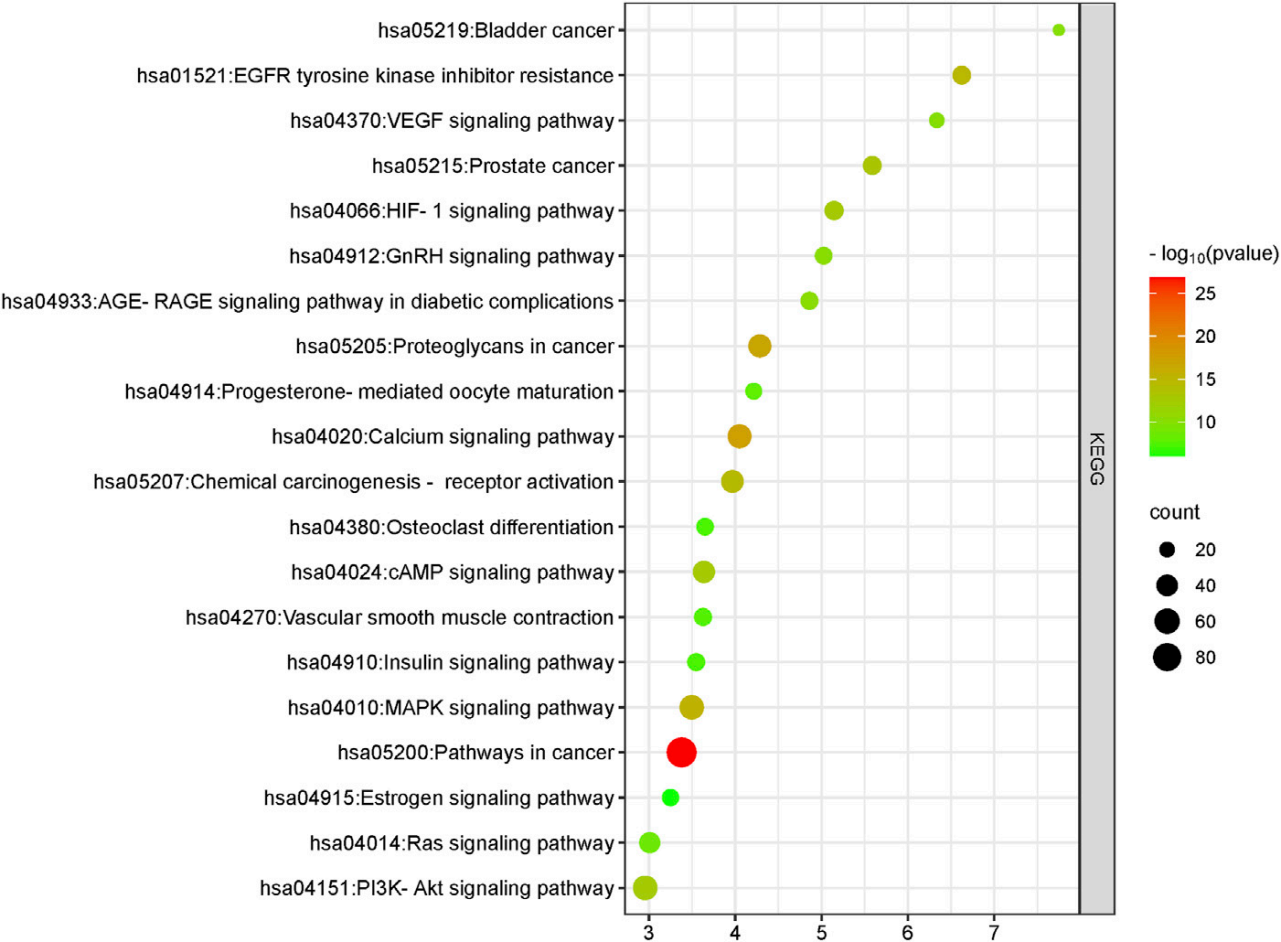
KEGG pathway analysis of common genes. This plot was generated using the ClusterProfiler package in R. All pathways depicted have a significant *p*-value of <0.05.

Our primary goal was to discern the potential interactions and roles of compounds present in *L. usitatissimum* within the intricate molecular landscape of ovarian cancer. To achieve this, we conducted a comprehensive KEGG pathway enrichment analysis (Figure 3; Supplementary File S1; Supplementary Table S4). This meticulous process illuminated significant involvement of these gene clusters in multiple KEGG pathways that are pivotal to our understanding of ovarian cancer’s molecular genesis and progression. Foremost among these is the “hsa05200: Pathways in cancer”, a broad and multifaceted pathway that offers an overarching view of various molecular interactions and dysregulations common in cancers. The identification of genes associated with the “hsa04151: PI3K-Akt signaling pathway” underscores the potential impact of *L. usitatissimum* compounds on cell survival, proliferation, and angiogenesis, all of which are key elements in tumor growth and metastasis. The enrichment in the “hsa05205: Proteoglycans in cancer” suggests a possible influence on cellular communication within the tumor microenvironment, while the presence of genes in the “hsa04012: ErbB signaling pathway” points towards implications in cell proliferation and differentiation.

Furthermore, with the detection of genes in the “hsa05207: Chemical carcinogenesis—receptor activation” and “hsa01521: EGFR tyrosine kinase inhibitor resistance” pathways, there’s an indication that the compounds may have a role in modulating the response to certain chemotherapeutic agents, potentially enhancing therapeutic efficacy. The significance of the “hsa05235: PD-L1 expression and PD-1 checkpoint pathway in cancer” cannot be understated in the era of immunotherapy, suggesting possible implications of *L. usitatissimum* in modulating immune responses in ovarian cancer. This pathway plays a pivotal role in immune regulation, where the interaction between programmed death-ligand 1 (PD-L1) expressed on cancer cells and programmed cell death protein 1 (PD-1) on immune cells can result in immune suppression, allowing tumor cells to evade immune surveillance. In the context of ovarian cancer, the significance of this pathway lies in its potential as a therapeutic target. High expression of PD-L1 by ovarian cancer cells has been associated with immune evasion and poor prognosis. Therefore, understanding and modulating this pathway hold promise for enhancing the immune response against ovarian cancer. In this study, we explore the potential of natural phytochemicals to influence the ‘hsa05235: PD-L1 expression and PD-1 checkpoint pathway in cancer,’ shedding light on novel avenues for ovarian cancer therapy (Pawłowska et al., 2019). Lastly, pathways like “hsa04066: HIF-1 signaling pathway” being highlighted showcases the potential of these compounds in influencing the cellular response to hypoxic conditions, a common feature of solid tumors.

Taken together, these findings not only amplify the potential therapeutic importance of *L. usitatissimum* compounds in ovarian cancer but also pave the way for subsequent studies aiming to unravel the full spectrum of molecular interactions and mechanisms. The elucidation of these pathways grants us a broader vista, one where the intersection of traditional botanical compounds and cutting-edge molecular oncology holds immense promise for future therapeutic interventions.

### 3.6 Compound-target-ovarian cancer network

**FIGURE 4:**
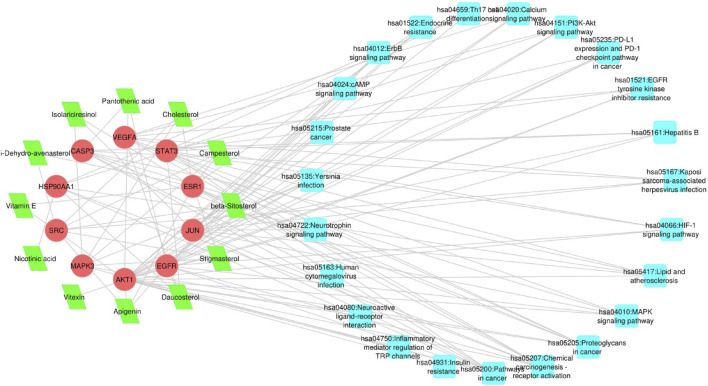
Compound-Target-Pathway Network: The nodes depicted in red represent genes, those in green symbolize active compounds, and the cyan nodes illustrate pathways primarily associated with the respective genes.

To delve deeper into the potential therapeutic mechanisms of *L. usitatissimum* compounds in the context of ovarian cancer, we developed a structured compound-target-disease network (Supplementary File S2: Supplementary Tables S1, S2). This network intricately maps the connections between active compounds derived from *L. usitatissimum*, their protein targets, and the KEGG pathways specifically related to ovarian cancer. Using PPI databases as our foundation, an expansive pathway-act-network was established. This framework vividly captures the complex interactions among various pathways, underscoring potential cross-talk and related associations significant to ovarian cancer. In Figure 4, the compound-target-pathway visualization reveals a complex network comprising 45 nodes and 111 edges. This intricate network unveils the extensive interplay between *L. usitatissimum* compounds and their connection to ovarian cancer. Of particular significance in this analysis is the pronounced synergy exhibited by these compounds, implying their combined efficacy in addressing ovarian cancer. This observation underscores the importance of exploring these compounds further for potential therapeutic interventions within the context of this disease. In summary, the compound-target-disease network is a crucial component of our study, enabling us to navigate the complex landscape of phytochemical interactions in the context of ovarian cancer therapy. It empowers us to make informed decisions about which phytochemicals to prioritize for further investigation, offers insights into potential molecular mechanisms, and supports the generation of hypotheses for future research. This network-based approach aligns with the systems pharmacology paradigm, enhancing our understanding of natural compounds’ therapeutic potential. By mapping out these interactions, we gain invaluable insights, suggesting that *L. usitatissimum* might offer a comprehensive approach to combating ovarian cancer and its intricate molecular landscape.

### 3.7 Survival analysis of hub genes

**FIGURE 5:**
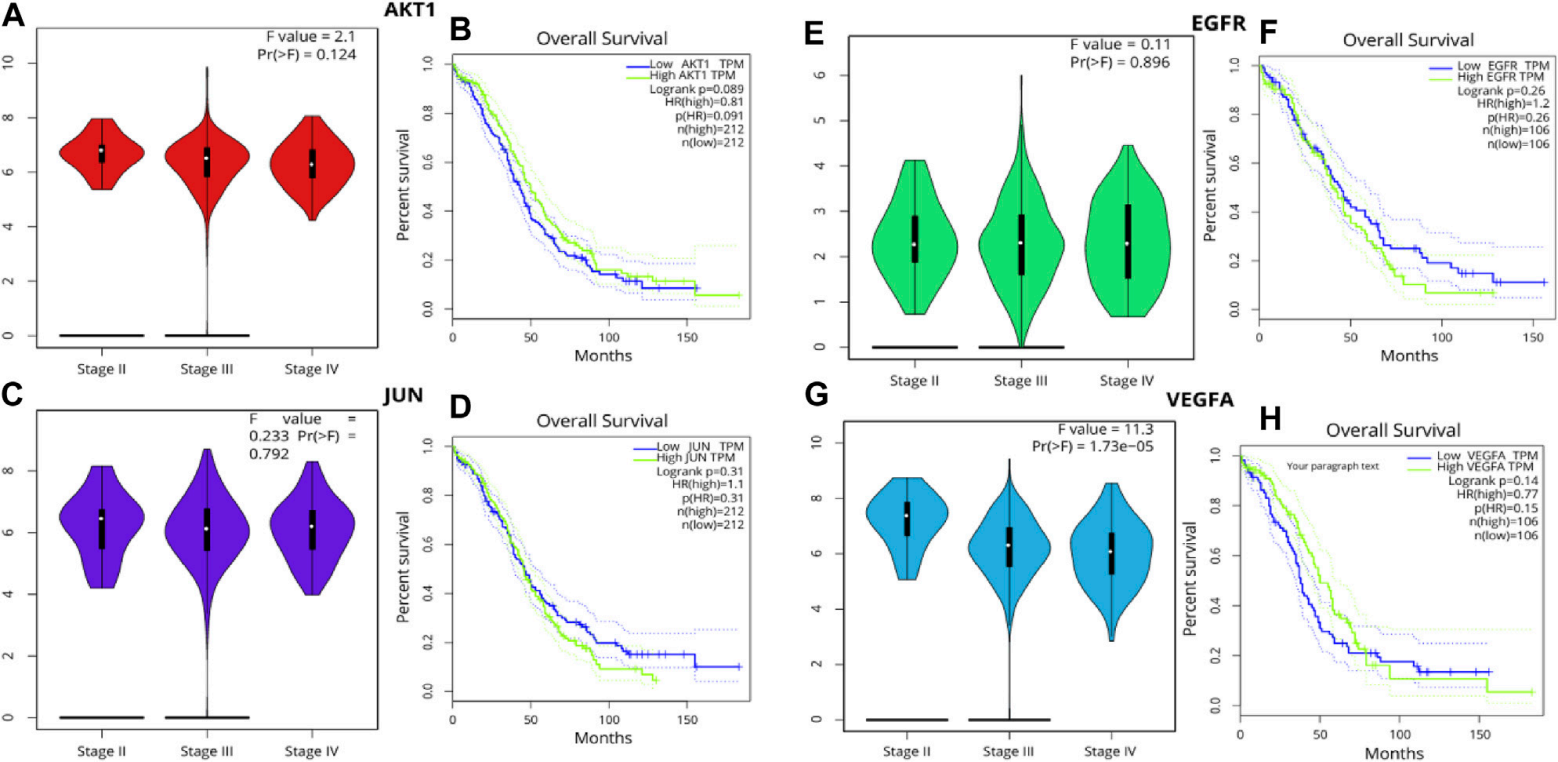
Pearson’s correlation analysis was leveraged to probe the associations among the hub genes **(A, B)** AKT1, **(C, D)** JUN **(E, F)** EGFR **(G, H)** VEGFA employing a significance threshold of *p* < 0.05. Genes exhibiting no correlation are denoted in blue, those with a negative correlation in red, and those revealing a positive correlation in green.

The Kaplan-Meier method remains a foundational statistical tool for projecting survival probabilities over time. This method provides invaluable insights into how factors—such as treatment modalities or genetic variations—affect survival outcomes. The Kaplan-Meier curve visually communicates these findings by illustrating the estimated survival rate across defined time intervals. This visual representation offers a comprehensive overview of the percentage of subjects remaining alive during the course of the study. Following the generation of Kaplan-Meier survival plots, the log-rank test, a pivotal statistical hypothesis tool, was employed. This test specializes in comparing the survival distributions among two or more distinct groups. In this context, it facilitated the comparison of survival outcomes between groups demarcated by high and low gene expression levels. In the midst of our analyses, certain genes demonstrated significant influence. However, the violin plot, showcasing the patient stage-wise F values, revealed critical insights. Specifically, AKT1 exhibited an F value of 2.1 and a Pr (>F) of 0.124. Similarly, EGFR presented an F value of 0.11 with a Pr (>F) of 0.896, JUN had an F value of 0.233 with a Pr (>F) of 0.792, and VEGFA stood out with an F value of 11.3 and a notably significant Pr (>F) of 1.73e-05. These statistical revelations accentuate the potential correlation between the expressions of these genes and ovarian cancer patient survival rates. Given the profound implications of these findings, the genes AKT1, EGFR, VEGFA, and JUN have been designated for further scrutiny, culminating in detailed molecular docking assessments (Figure 5).

### 3.8 Molecular docking analysis

**FIGURE 6:**
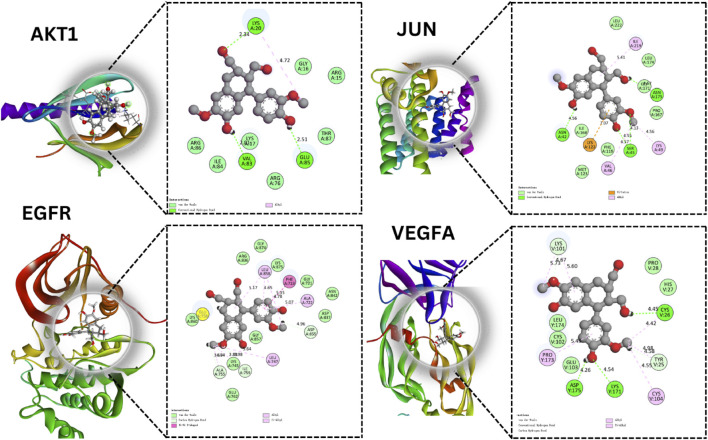
Molecular Docking Analysis of Selected Proteins. This figure showcases the molecular interactions of specific compounds with four key proteins AKT1, JUN, EGFR, and VEGFA.

In our exploration focused on ovarian cancer therapeutics, molecular docking studies were undertaken to understand the potential interactions between identified bioactive compounds and critical proteins implicated in ovarian cancer pathogenesis (Figure 6). In our study, we utilized the CASTp tool to identify and assess the binding pockets of four target proteins, namely, AKT1, EGFR, VEGFA, and JUN. The CASTp scores provided valuable insights into the characteristics of these binding pockets, with computed values serving as indicators of their area and volume. For AKT1, we found an area of 499.2 Å^2^ and a volume of 300.8 Å³, while EGFR exhibited an area of 450,1 Å^2^ and a volume of 320.7 Å³. VEGFA displayed an area of 480.0 Å^2^ and a volume of 310.4 Å³, and JUN had an area of 490.9 Å^2^ and a volume of 315.5 Å³.Following the CASTp analysis, we proceeded to perform molecular docking experiments using PyRx. In this phase, we defined docking grid coordinates to precisely target these binding pockets. For AKT1, we set the grid at X: −5.0, Y: −12.0, Z: −23.0, with an exhaustiveness parameter of 8. Similarly, for EGFR, we placed the grid at X: 20.0, Y: 18.0, Z: 30.0, also with an exhaustiveness of 8. VEGFA’s grid was positioned at X: −3.0, Y: 15.0, Z: −25.0, with an exhaustiveness of 8, while JUN’s grid coordinates were X: 22.0, Y: 20.0, Z: 32.0, also with an exhaustiveness of 8. These methodologies allowed us to gain valuable insights into potential binding interactions between our target proteins and ligands, ultimately contributing to a deeper understanding of their functional roles and potential applications in drug discovery or therapeutic development. The AKT1 protein stands at the crossroads of multiple cellular pathways, dictating cell survival, growth, and proliferation. Our findings that Compound1 formed hydrogen bonds with residues Lys A:20, Glu A:85, and Val A:83 of AKT1 indicate that this compound might be able to modulate AKT1 activity. Given the importance of the AKT signaling pathway in various malignancies, including ovarian cancer, this interaction promises potential therapeutic benefits. The JUN protein’s role in oncogenesis, especially in cell proliferation and differentiation, is well-established. The fact that Compound1 interacted with JUN residues Asn A:175, Asn A:42, and Ser:45 suggests the compound’s capacity to impede JUN’s function. This might translate into a suppression of its oncogenic effects, opening avenues for novel therapeutic strategies.

With the EGFR protein, a significant contributor to cancer proliferation and survival due to its tyrosine kinase activity, Isolariciresinol interaction at the Asp A:855 residue holds therapeutic significance. Inhibiting EGFR signaling could halt the malignant progression of ovarian cancer cells. Finally, VEGFA is instrumental in fostering angiogenesis, the very process granting tumors their nutrient supply. Isolariciresinol binding interactions with VEGFA residues - Cys V:256, Asp Y:175, and Lys A:171 - could signify a blockade in angiogenic pathways. By starving the tumor of its essential nutrient supply, this interaction holds the potential to significantly limit tumor growth and metastasis (Table 2).

**TABLE 2:** Binding affinity and RMSD values of active proteins against active constituents.

Target receptor	Compounds I’ds	Compounds name	Docking score	RMSD	Binding affinity
AKT1	160,521	Isolariciresinol	−14.94 kcal/mol	1.86	−13.04 kcal/mol
	14,985	Vitamin E	−16.67 kcal/mol	1.04	−15.57 kcal/mol
	985	Palmitic Acid	−15.52 kcal/mol	0.69	−15.52 kcal/mol
	493,570	Riboflavin	−15.04 kcal/mol	2.02	−14.14 kcal/mol
	6,613	Pantothenic Acid	−14.26 kcal/mol	2.81	−12.61 kcal/mol
EGFR	160,521	Isolariciresinol	−15.68 kcal/mol	0.94	−14.81 kcal/mol
	222,284	Beta-Sitosterol	−15.41 kcal/mol	1.96	−13.01 kcal/mol
	493,570	Riboflavin	−14.22 kcal/mol	1.62	−13.72 kcal/mol
	44,263,331	5-Dehydroavenasterol	−14.01 kcal/mol	2.86	−12.41 kcal/mol
	173,183	Campestrol	−12.41 kcal/mol	1.19	−11.92 kcal/mol
JUN	5,280,443	Apigenin	−14.91 kcal/mol	2.05	−13.11 kcal/mol
	160,521	Isolariciresinol	−13.08 kcal/mol	1.76	−12.51 kcal/mol
	493,570	Riboflavin	−12.97 kcal/mol	0.71	−11.42 kcal/mol
	14,985	Vitamin E	−12.67 kcal/mol	2.16	−11.72 kcal/mol
	6,613	Pantothenic Acid	−12.18 kcal/mol	1.87	−11.71 kcal/mol
VEGFA	6,613	Pantothenic Acid	−16.83 kcal/mol	1.09	−15.11 kcal/mol
	493,570	Riboflavin	−14.16 kcal/mol	1.95	−13.01 kcal/mol
	44,263,331	5-Dehydroavenasterol	−13.08 kcal/mol	1.35	−12.17 kcal/mol
	160,521	Isolariciresinol	−11.04 kcal/mol	1.64	−10.41 kcal/mol
	5,280,443	Apigenin	−10.82 kcal/mol	1.36	−9.21 kcal/mol

While these findings spotlight Isolariciresinol diverse therapeutic potential against ovarian cancer, they also underline the complexity of cancer as a disease. It is imperative to further validate these interactions through rigorous *in-vitro* and *in-vivo* studies. Given the preliminary insights, Compound1 is poised as a multi-target agent that might revolutionize ovarian cancer therapeutics.

### 3.9 MD simulation

**FIGURE 7:**
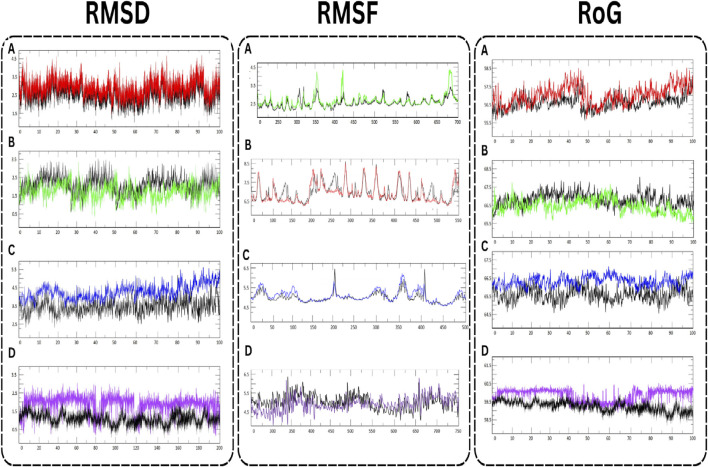
MD simulation of target proteins with active compounds. **(A)** AKT1 **(B)** JUN **(C)** EGFR, and **(D)** VEGFA.

In the comprehensive MD simulations undertaken, distinctive behaviors of specific proteins in their interaction with active compounds were observed (Figure 7). The RMSD values, which offer insights into the stability of protein-ligand interactions, showed variances among the proteins. For AKT1 in complex with Isolariciresinol, the RMSD measurements hovered around an average of 3 Å (angstroms), suggesting a consistent interaction pattern with its respective ligand. On the other hand, both JUN and VEGFA displayed somewhat tighter interactions, as evidenced by their RMSD values nearing 2 Å. The EGFR protein was somewhat more dynamic, with RMSD values oscillating between 3.5 and 4.0 Å, indicating a potentially broader range of conformational changes during its interaction. Further insight was gathered from the RMSF analysis, which provides a lens into the flexibility and motion of specific regions within the protein structures. Both JUN and EGFR showcased heightened fluctuations, hinting at potential areas within these proteins that may experience greater flexibility or dynamic changes when bound to ligands. Interestingly, AKT1 displayed a unique behavior, with its central region showing pronounced fluctuations, suggesting this region might undergo significant conformational changes during ligand interactions. Lastly, the RoG analysis served as a testament to the overall stability of the protein-ligand complexes. Across the board, all complexes maintained stable RoG values, implying that these complexes sustain a consistent and compact structural formation throughout the simulation duration. This stability, coupled with the intricate interactions observed, underscores the potential of these proteins and their ligands as significant subjects for further investigation in molecular research. The study successfully confirmed the potential inhibitory effects of the selected plant-derived molecules, AKT1, JUN, EGFR, and VEGFA proteins. Their observed stability and minimal flexibility make these complexes promising candidates for further research in ovarian cancer therapy. In summary, this provides a scientific foundation for understanding the potential of *L. usitatissimum* as viable treatment options for ovarian cancer. The integration of network pharmacology and bioinformatics allows for the identification of critical molecular pathways and interactions related to ovarian cancer, thus offering new avenues for therapeutic interventions. Though the findings are substantiated through molecular docking and MD simulations, further validation via *in vitro* and *in vivo* studies remains essential.

### 3.10 Binding free energy calculation using

**FIGURE 8:**
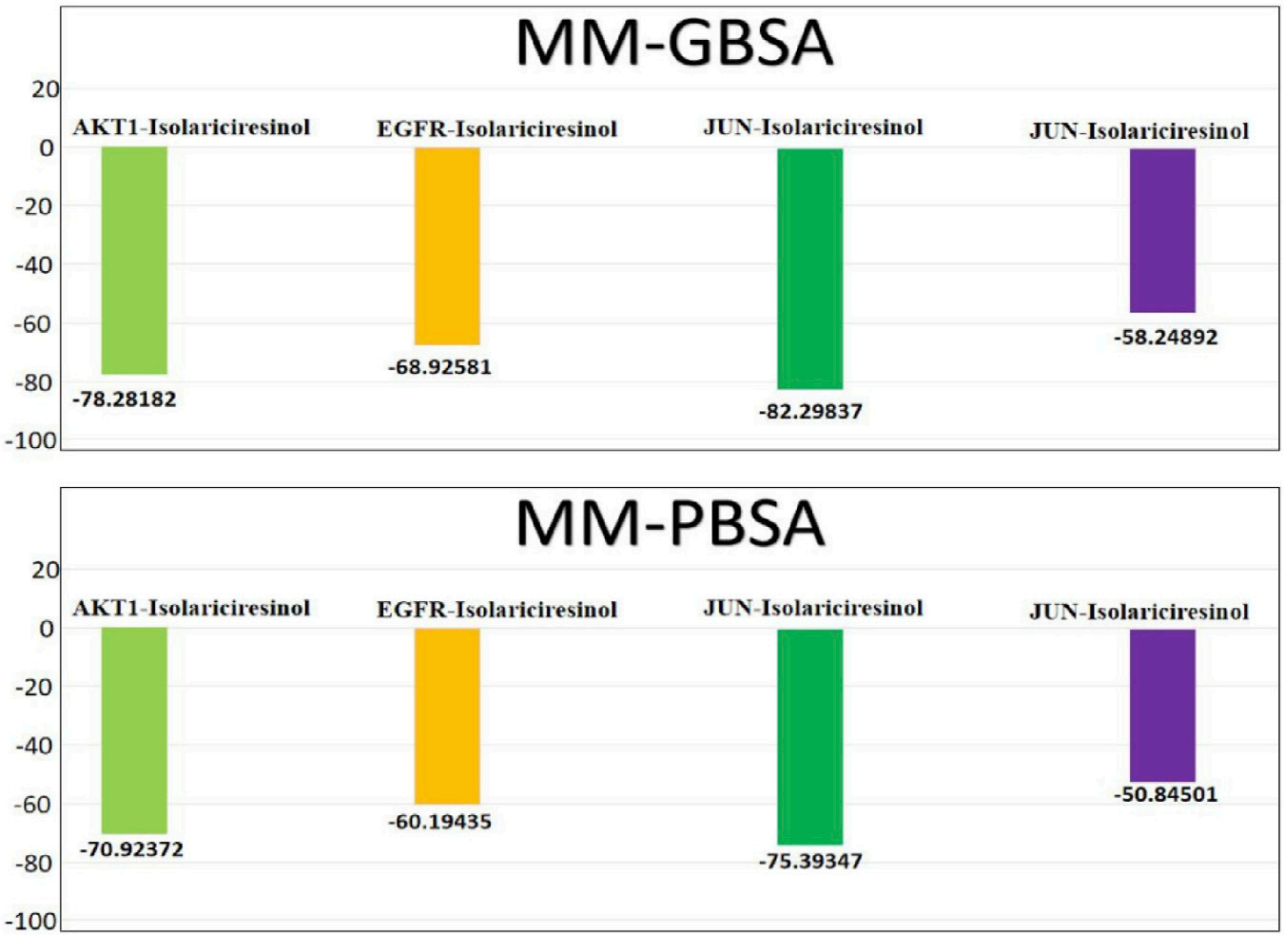
Binding free energies (kcal/mol) and the individual energetic terms for the systems using the MMGBSA and MMPBSA method.

Molecular Mechanics energies combined with the Generalized Born and Surface Area continuum solvation (MM/GBSA) and Molecular Mechanics energies combined with the Poisson-Boltzmann Surface Area continuum solvation (MM/PBSA) are powerful post-docking computational approaches (Figure 8). They are widely employed in the drug design arena to estimate the binding free energy between a ligand and its target protein. These methodologies not only provide a quantitative measure of ligand binding affinity but also offer qualitative insights into the potential interaction modes and energetic contributions of individual residues at the binding interface. In our recent analysis, the binding affinities of the molecule Isolariciresinol with genes AKT1, EGFR, JUN, and VEGFA were scrutinized using both MM/GBSA and MM/PBSA methodologies. The MM/GBSA analysis divulged that the binding of Isolariciresinol to AKT1, EGFR, JUN, and VEGFA resulted in ΔG values of −78.28, −68.92, −82.29, and −58.24 kcal/mol, respectively. This suggests a strong and energetically favorable interaction, particularly with the JUN protein. Subsequently, the MM/PBSA analysis corroborated these findings, although with marginally varied magnitudes. The ΔG values for AKT1, EGFR, JUN, and VEGFA were recorded as −70.92, −60.19, −75.39, and −50.84 kcal/mol, respectively. Notably, both methods indicated the highest binding affinity with the JUN protein, reinforcing its potential as a prime therapeutic target. It is evident from the MM/GBSA and MM/PBSA results that Isolariciresinol exhibits promising binding potential with all four genes, though its affinity with JUN stands out prominently. Such analyses are pivotal in the drug discovery process, aiding in refining lead compounds and steering them closer to potential drug candidates.

## 4 Discussion

Ovarian cancer, often dubbed as the “silent killer” of women, represents a severe threat in the realm of gynecological malignancies (Jayson et al., 2014). Its subtle onset and propensity for late-stage diagnosis have made its management a formidable challenge. While advancements in medical sciences have considerably improved diagnosis and treatment modalities, there’s an unequivocal demand for more targeted, less toxic, and highly effective therapeutic strategies. Historically, plants have been an indispensable source of therapeutic agents. The ancient medicinal herb, *L. usitatissimum*, commonly known as flaxseed, is a case in point (Mani et al., 2011). With its rich repository of bioactive compounds and health benefits, flaxseed’s potential role in ameliorating or even combating ovarian cancer demands rigorous scientific exploration.

Our investigative journey into *L. usitatissimum* has been both illuminating and promising. From the array of compounds identified, Apigenin, Vitamin E, Palmitic acid, Riboflavin, Isolariciresinol, 5-Dehydro-avenasterol, Cholesterol, Pantothenic acid, Nicotinic acid, Campesterol, Beta-Sitosterol, Stigmasterol, Daucosterol, and Vitexin have emerged as stellar candidates. Their molecular structures, pharmacokinetic properties, and potential interactions with cellular pathways suggest a drug-like potential, which could be harnessed for therapeutic interventions.

A deeper foray into the molecular framework unraveled an intricate dance of 494 common genes. These genes, potentially influenced by flaxseed’s bioactive compounds, painted a comprehensive picture of the potential cellular impacts. Among these genes, proteins such as AKT1, SRC, VEGFA, MAPK3, EGFR, HSP90AA1, STAT3, JUN, CASP3, and ESR1 rose to prominence, signaling their pivotal role in ovarian cancer’s biology. The survival analysis further refined our focus, pinpointing AKT1, EGFR, VEGFA, and JUN as the most crucial determinants. AKT1, a serine/threonine kinase, is an integral part of the PI3K/AKT signaling pathway, often found dysregulated in ovarian tumors. Its activation is crucial for several cellular processes, including proliferation, survival, and angiogenesis, making it a potential therapeutic target in combatting tumor progression (Meng et al., 2006). Parallelly, the Epidermal Growth Factor Receptor (EGFR) is frequently overexpressed in ovarian cancer and is associated with poor prognosis. This receptor tyrosine kinase plays a pivotal role in cell growth, differentiation, and migration, and its aberrant signaling often leads to uncontrolled tumor growth and metastasis (Sheng and Liu, 2011). Vascular Endothelial Growth Factor A (VEGFA) is another key player, mainly responsible for promoting angiogenesis, a fundamental process that provides the tumor with the necessary blood supply. Elevated levels of VEGFA in ovarian tumors have been linked to advanced disease stages and reduced survival rates (Jang et al., 2017). Lastly, the proto-oncogene JUN, a component of the AP-1 transcription factor complex, is involved in regulating various cellular activities like cell cycle progression, apoptosis, and cell differentiation. In the context of ovarian cancer, JUN is often upregulated, driving the transcription of genes that promote tumor growth and invasion. Together, these four molecular giants shape the landscape of ovarian cancer progression and offer potential avenues for targeted therapies (Neyns et al., 1996).

The KEGG pathway analysis reveals that the identified genes are predominantly associated with several signaling cascades with implications for ovarian cancer. Foremost among them is the PI3K-Akt signaling pathway, a crucial axis frequently hyperactivated in ovarian malignancies, known to promote tumor survival, proliferation, and angiogenesis (Ediriweera et al., 2019). This is complemented by the VEGF signaling pathway, responsible for angiogenesis, which is often upregulated in ovarian tumors, suggesting a pivotal role in tumoral blood supply and nutrition (He et al., 2016). The HIF-1 signaling pathway further solidifies the tumor’s adaptability under hypoxic conditions, characteristic of rapidly proliferating malignancies (Ai et al., 2016). The intricacies of hormonal impact on ovarian tumorigenesis are also highlighted. The Estrogen signaling pathway indicates estrogen’s central role in ovarian cancer’s progression, and the Progesterone-mediated oocyte maturation signaling points towards hormonal imbalances possibly contributing to tumor genesis (Ribeiro and Freiman, 2014). While the Prostate Cancer and Bladder Cancer pathways primarily define other malignancies, their molecular mechanisms might intersect with ovarian tumorigenesis, suggesting potential shared therapeutic targets. Lastly, the cAMP signaling pathway and Osteoclast differentiation might signify the intricate balance between cell growth and death and potential metastatic pathways in ovarian cancer. Collectively, these findings underscore the multi-faceted molecular landscape of ovarian cancer and indicate that targeting genes within these pathways could offer a comprehensive therapeutic strategy, potentially halting tumor progression from multiple angles.

Given the central roles these proteins play in cellular functions and pathologies, the potential of *L. usitatissimum’s* bioactive compounds to modulate their activity could reshape our therapeutic strategies. The multifaceted actions of these compounds, spanning from modulation of cell signaling pathways to influencing transcriptional activities, imply a holistic therapeutic impact, much needed in the intricate scenario of ovarian cancer. While our research provides valuable insights into the potential mechanisms underlying the effects of indigenous plants on ovarian cancer, it is not without its limitations. First and foremost, the initial results presented here are primarily computational and thus require further validation through *in vitro* and *in vivo* experimental studies. Such studies would help confirm the pharmacokinetic and pharmacodynamic properties of the identified active compounds, and their actual efficacy in modulating ovarian cancer and its associated complications. Secondly, our network pharmacology analysis relies on existing databases of traditional remedies and target genes, which are not exhaustive. Future efforts to expand and update these databases could substantially improve the accuracy and reliability of our models. This is particularly relevant given the vast array of traditional plant remedies that have yet to be fully characterized, and the continually evolving understanding of gene functions and interactions.

Thirdly, while our study employed both network pharmacology and molecular docking techniques, it still fell short of providing a complete picture of the therapeutic potential of phytochemicals against ovarian cancer. These methods primarily offer a macroscopic view of possible compound-target interactions and pathways but do not fully elucidate the complex pharmacological actions, such as synergistic or antagonistic effects, that these compounds may exhibit in a biological setting. Lastly, our research does not address the potential influence of patient-specific variables, such as genetics or pre-existing conditions, which could significantly impact the effectiveness of plant-based treatments for ovarian cancer. Nor does it consider the practical challenges associated with converting these active compounds into clinically viable formulations. Hence, a comprehensive and multidisciplinary approach that integrates pharmacology, bioinformatics, experimental biology, and clinical trials becomes imperative. This approach is crucial for gaining a holistic understanding of how L. usitatissimum can be effectively harnessed to combat ovarian cancer.

## 5 Conclusion

In conclusion, ovarian cancer, a formidable adversary in women’s health, demands innovative therapeutic strategies that are both efficacious and safe. Our comprehensive research highlights the promising potential of *L. usitatissimum*, a natural remedy, in addressing the healthcare challenges posed by ovarian cancer. Through the application of advanced techniques such as data mining, network pharmacology, and molecular docking, we have unveiled the intricate molecular mechanisms that underlie the therapeutic efficacy of *L. usitatissimum* in combatting ovarian cancer. Hub genes, including AKT1, JUN, EGFR, and VEGFA, have emerged as significant contributors to these mechanisms. Moreover, compounds such as Apigenin, Isolariciresinol, and Beta-Sitosterol have exhibited notable effects on modulating the expression patterns of these genes. Furthermore, our rigorous analysis, including Kaplan–Meier survival analysis, suggests that these genes could serve as valuable biomarkers for ovarian cancer diagnosis and prognosis. The molecular docking studies, complemented by MMGBSA and MMPBSA analyses, not only validate the strong binding affinities of these compounds but also underscore their stability and robust affinity towards their respective binding sites. While these findings illuminate a promising avenue for ovarian cancer treatment harnessing the virtues of *L. usitatissimum*, it remains quintessential to further validate these results through rigorous *in vivo* and *in vitro* experiments. Such endeavors will pave the way for a holistic understanding of the pharmacokinetics and biosafety profiles, potentially elevating *L. usitatissimum* from a traditional remedy to a mainstream therapeutic for ovarian cancer.

## Data Availability

The original contributions presented in the study are included in the article/Supplementary Material, further inquiries can be directed to the corresponding authors.

## References

[B1] Abbas S. Q. Muhammad I. Wu J.-J. Yan S.-K. Ali F. Majid M. (2022). Metals-triggered compound CDPDP exhibits anti-arthritic behavior by downregulating the inflammatory cytokines, and modulating the oxidative storm in mice models with extensive ADMET, docking and simulation studies. Front. Pharmacol. 13, 1053744. 10.3389/fphar.2022.1053744 36506587 PMC9727203

[B2] Adolphe J. L. Whiting S. J. Juurlink B. H. Thorpe L. U. Alcorn J. (2010). Health effects with consumption of the flax lignan secoisolariciresinol diglucoside. Br. J. Nutr. 103 (7), 929–938. 10.1017/S0007114509992753 20003621

[B3] Ai Z. Lu Y. Qiu S. Fan Z. (2016). Overcoming cisplatin resistance of ovarian cancer cells by targeting HIF-1-regulated cancer metabolism. Cancer Lett. 373 (1), 36–44. 10.1016/j.canlet.2016.01.009 26801746 PMC4769873

[B4] Aqeel M. T. Rahman N. U. Khan A. U. Khan M. T. Ashraf Z. ul Hassan S. S. (2023). Cardioprotective effect of 2-methoxy phenol derivatives against oxidative stress-induced vascular complications: an integrated *in vitro*, *in silico*, and *in vivo* investigation. Biomed. Pharmacother. 165, 115240. 10.1016/j.biopha.2023.115240 37531779

[B5] Asghar A. Qasim M. Noor F. Ashfaq U. A. Tahir ul Qamar M. Masoud M. S. (2023). Systematic elucidation of the multi-target pharmacological mechanism of Terminalia arjuna against congestive cardiac failure via network pharmacology and molecular modelling approaches. Nat. Prod. Res., 1–8. 10.1080/14786419.2023.2252565 37665010

[B6] Basavarajappa G. M. Rehman A. Shiroorkar P. N. Sreeharsha N. Anwer M. K. Aloufi B. (2023). Therapeutic effects of Crataegus monogyna inhibitors against breast cancer. Front. Pharmacol. 14, 1187079. 10.3389/fphar.2023.1187079 37180727 PMC10174464

[B7] Batool S. Javed M. R. Aslam S. Noor F. Javed H. M. F. Seemab R. (2022a). Network pharmacology and bioinformatics approach reveals the multi-target pharmacological mechanism of fumaria indica in the treatment of liver cancer. Pharm. (Basel, Switz. 15 (6), 654. 10.3390/ph15060654 PMC9229061 35745580

[B8] Batool S. Javed M. R. Aslam S. Noor F. Javed H. M. F. Seemab R. (2022b). Network pharmacology and bioinformatics approach reveals the multi-target pharmacological mechanism of Fumaria indica in the treatment of liver cancer. Pharmaceuticals 15 (6), 654. 10.3390/ph15060654 35745580 PMC9229061

[B9] Bray F. Ferlay J. Soerjomataram I. Siegel R. L. Torre L. A. Jemal A. (2018). Global cancer statistics 2018: GLOBOCAN estimates of incidence and mortality worldwide for 36 cancers in 185 countries. CA a cancer J. Clin. 68 (6), 394–424. 10.3322/caac.21492 30207593

[B10] Cibula D. Zikan M. Dusek L. Majek O. (2011). Oral contraceptives and risk of ovarian and breast cancers in BRCA mutation carriers: a meta-analysis. Expert Rev. anticancer Ther. 11 (8), 1197–1207. 10.1586/era.11.38 21916573

[B11] Dallakyan S. Olson A. J. (2015). Small-molecule library screening by docking with PyRx. Chemical biology. Springer, 243–250. 10.1007/978-1-4939-2269-7_19 25618350

[B12] Ediriweera M. K. Tennekoon K. H. Samarakoon S. R. (Editors) (2019). “Role of the PI3K/AKT/mTOR signaling pathway in ovarian cancer: biological and therapeutic significance,” Seminars in cancer biology (Elsevier). 10.1016/j.semcancer.2019.05.012 31128298

[B13] Flower G. Fritz H. Balneaves L. G. Verma S. Skidmore B. Fernandes R. (2014). Flax and breast cancer: a systematic review. Integr. Cancer Ther. 13 (3), 181–192. 10.1177/1534735413502076 24013641

[B14] Fritsche K. L. (2015). The science of fatty acids and inflammation. Adv. Nutr. 6 (3), 293S–301S. 10.3945/an.114.006940 25979502 PMC4424767

[B15] Gfeller D. Grosdidier A. Wirth M. Daina A. Michielin O. Zoete V. (2014). SwissTargetPrediction: a web server for target prediction of bioactive small molecules. Nucleic Acids Res. 42 (W1), W32–W38. 10.1093/nar/gku293 24792161 PMC4086140

[B16] Goff B. A. Mandel L. Muntz H. G. Melancon C. H. (2000). Ovarian carcinoma diagnosis: results of a national ovarian cancer survey. Cancer. Interdiscip. Int. J. Am. Cancer Soc. 89 (10), 2068–2075. 10.1002/1097-0142(20001115)89:10<2068::aid-cncr6>3.0.co;2-z 11066047

[B17] Hamosh A. Scott A. F. Amberger J. Valle D. McKusick V. A. (2000). Online Mendelian inheritance in man (OMIM). Hum. Mutat. 15 (1), 57–61. 10.1002/(SICI)1098-1004(200001)15:1<57::AID-HUMU12>3.0.CO;2-G 10612823

[B18] He Z. Chen A. Y. Rojanasakul Y. Rankin G. O. Chen Y. C. (2016). Gallic acid, a phenolic compound, exerts anti-angiogenic effects via the PTEN/AKT/HIF-1α/VEGF signaling pathway in ovarian cancer cells. Oncol. Rep. 35 (1), 291–297. 10.3892/or.2015.4354 26530725 PMC4699619

[B19] Jang K. Kim M. Gilbert C. A. Simpkins F. Ince T. A. Slingerland J. M. (2017). VEGFA activates an epigenetic pathway upregulating ovarian cancer‐initiating cells. EMBO Mol. Med. 9 (3), 304–318. 10.15252/emmm.201606840 28179359 PMC5331266

[B20] Jayson G. C. Kohn E. C. Kitchener H. C. Ledermann J. A. (2014). Ovarian cancer. Lancet 384 (9951), 1376–1388. 10.1016/S0140-6736(13)62146-7 24767708

[B21] Khan K. Alhar M. S. O. Abbas M. N. Abbas S. Q. Kazi M. Khan S. A. (2022a). Integrated bioinformatics-based subtractive genomics approach to decipher the therapeutic drug target and its possible intervention against brucellosis. Bioengineering 9 (11), 633. 10.3390/bioengineering9110633 36354544 PMC9687753

[B22] Khan N. U. Qazi N. G. Khan A. U. Ali F. Hassan S. S. Bungau S. (2022b). Anti-diabetic activity of brucine in streptozotocin-induced rats: *in silico*, *in vitro*, and *in vivo* studies. ACS omega 7 (50), 46358–46370. 10.1021/acsomega.2c04977 36570195 PMC9774404

[B23] Kim S. Chen J. Cheng T. Gindulyte A. He J. He S. (2021). PubChem in 2021: new data content and improved web interfaces. Nucleic Acids Res. 49 (D1), D1388–D1395. 10.1093/nar/gkaa971 33151290 PMC7778930

[B24] Kolarevic A. Yancheva D. Kocic G. Smelcerovic A. (2014). Deoxyribonuclease inhibitors. Eur. J. Med. Chem. 88, 101–111. 10.1016/j.ejmech.2014.07.040 25042005

[B25] Kouranov A. Xie L. de la Cruz J. Chen L. Westbrook J. Bourne P. E. (2006). The RCSB PDB information portal for structural genomics. Nucleic acids Res. 34 (1), D302–D305. 10.1093/nar/gkj120 16381872 PMC1347482

[B26] Kuhn M. von Mering C. Campillos M. Jensen L. J. Bork P. (2007). STITCH: interaction networks of chemicals and proteins. Nucleic Acids Res. 36 (1), D684–D688. 10.1093/nar/gkm795 18084021 PMC2238848

[B27] Kurman R. J. Shih I.-M. (2010). The origin and pathogenesis of epithelial ovarian cancer-a proposed unifying theory. Am. J. Surg. pathology 34 (3), 433–443. 10.1097/PAS.0b013e3181cf3d79 PMC2841791 20154587

[B28] Mani U. V. Mani I. Biswas M. Kumar S. N. (2011). An open-label study on the effect of flax seed powder (Linum usitatissimum) supplementation in the management of diabetes mellitus. J. Diet. Suppl. 8 (3), 257–265. 10.3109/19390211.2011.593615 22432725

[B29] Meng Q. Xia C. Fang J. Rojanasakul Y. Jiang B.-H. (2006). Role of PI3K and AKT specific isoforms in ovarian cancer cell migration, invasion and proliferation through the p70S6K1 pathway. Cell. Signal. 18 (12), 2262–2271. 10.1016/j.cellsig.2006.05.019 16839745

[B30] Mirza M. R. Monk B. J. Herrstedt J. Oza A. M. Mahner S. Redondo A. (2016). Niraparib maintenance therapy in platinum-sensitive, recurrent ovarian cancer. N. Engl. J. Med. 375 (22), 2154–2164. 10.1056/NEJMoa1611310 27717299

[B31] Mohanraj K. Karthikeyan B. S. Vivek-Ananth R. Chand R. Aparna S. Mangalapandi P. (2018). IMPPAT: a curated database of Indian medicinal plants. phytochemistry Ther. 8 (1), 1–17. 10.1038/s41598-018-22631-z PMC5847565 29531263

[B32] Morris D. H. (2007). Flax: a health and nutrition primer. Flax Council of Canada.

[B33] Noor F. Rehman A. Ashfaq U. A. Saleem M. H. Okla M. K. Al-Hashimi A. (2022). Integrating network pharmacology and molecular docking approaches to decipher the multi-target pharmacological mechanism of *Abrus precatorius* L. Acting on diabetes. Pharm. (Basel, Switz. 15 (4), 414. 10.3390/ph15040414 PMC9029140 35455411

[B34] Nakamura K. Shimura N. Otabe Y. Hirai-Morita A. Nakamura Y. Ono N. (2013). KNApSAcK-3D: a three-dimensional structure database of plant metabolites. Plant Cell. Physiol. 54 (2), e4–e. 10.1093/pcp/pcs186 23292603

[B35] Neyns B. Vermeij J. Bourgain C. Vandamme B. Amfo K. Lissens W. (1996). Expression of the jun family of genes in human ovarian cancer and normal ovarian surface epithelium. Oncogene 12 (6), 1247–1257. 8649827

[B36] Noor F. Tahir Ul Qamar M. Ashfaq U. A. Albutti A. Alwashmi A. S. S. Aljasir M. A. (2022). Network pharmacology approach for medicinal plants: review and assessment. Pharm. (Basel, Switz. 15 (5), 572. 10.3390/ph15050572 PMC9143318 35631398

[B37] Noor F. Asif M. Ashfaq U. A. Qasim M. Tahir Ul Qamar M. (2023). Machine learning for synergistic network pharmacology: a comprehensive overview. Brief. Bioinform 24 (3), bbad120. 10.1093/bib/bbad120 37031957

[B38] Pawłowska A. Suszczyk D. Okła K. Barczyński B. Kotarski J. Wertel I. (2019). Immunotherapies based on PD-1/PD-L1 pathway inhibitors in ovarian cancer treatment. Clin. Exp. Immunol. 195 (3), 334–344. 10.1111/cei.13255 30582756 PMC6378380

[B39] Pettersen E. F. Goddard T. D. Huang C. C. Couch G. S. Greenblatt D. M. Meng E. C. (2004). UCSF Chimera—a visualization system for exploratory research and analysis. J. Comput. Chem. 25 (13), 1605–1612. 10.1002/jcc.20084 15264254

[B40] Prat J. (2012). Ovarian carcinomas: five distinct diseases with different origins, genetic alterations, and clinicopathological features. Virchows Arch. 460 (3), 237–249. 10.1007/s00428-012-1203-5 22322322

[B41] Qasim M. Abdullah M. Ali Ashfaq U. Noor F. Nahid N. Alzamami A. (2023). Molecular mechanism of Ferula asafoetida for the treatment of asthma: network pharmacology and molecular docking approach. Saudi J. Biol. Sci. 30 (2), 103527. 10.1016/j.sjbs.2022.103527 36568408 PMC9772567

[B42] Rehman A. Ashfaq U. A. Javed M. R. Shahid F. Noor F. Aslam S. (2022). The Screening of phytochemicals against NS5 Polymerase to treat Zika Virus infection: integrated computational based approach. Comb. Chem. High Throughput Screen. 25 (4), 738–751. 10.2174/1386207324666210712091920 34254908

[B43] Rehman A. Fatima I. Wang Y. Tong J. Noor F. Qasim M. (2023). Unveiling the multi-target compounds of Rhazya stricta: discovery and inhibition of novel target genes for the treatment of clear cell renal cell carcinoma. Comput. Biol. Med. 165, 107424. 10.1016/j.compbiomed.2023.107424 37717527

[B44] Reid B. M. Permuth J. B. Sellers T. A. (2017). Epidemiology of ovarian cancer: a review. Cancer Biol. Med. 14 (1), 9–32. 10.20892/j.issn.2095-3941.2016.0084 28443200 PMC5365187

[B45] Ribeiro J. R. Freiman R. N. (2014). Estrogen signaling crosstalk: implications for endocrine resistance in ovarian cancer. J. steroid Biochem. Mol. Biol. 143, 160–173. 10.1016/j.jsbmb.2014.02.010 24565562 PMC4127339

[B46] Ru J. Li P. Wang J. Zhou W. Li B. Huang C. (2014). TCMSP: a database of systems pharmacology for drug discovery from herbal medicines. J. Cheminform 6, 13. 10.1186/1758-2946-6-13 24735618 PMC4001360

[B47] Safran M. Dalah I. Alexander J. Rosen N. Iny Stein T. Shmoish M. (2010). GeneCards Version 3: the human gene integrator. Database 2010, baq020. 10.1093/database/baq020 20689021 PMC2938269

[B48] Saggar J. K. Chen J. Corey P. Thompson L. U. (2010). Dietary flaxseed lignan or oil combined with tamoxifen treatment affects MCF‐7 tumor growth through estrogen receptor‐and growth factor‐signaling pathways. Mol. Nutr. food Res. 54 (3), 415–425. 10.1002/mnfr.200900068 19904759

[B49] Shams ul Hassan S. Abbas S. Q. Hassan M. Jin H.-Z. (2022). Computational exploration of anti-cancer potential of guaiane dimers from Xylopia vielana by targeting B-RAF kinase using chemo-informatics, molecular docking, and MD simulation studies. Anti-Cancer Agents Med. Chem. Former. Curr. Med. Chemistry-Anti-Cancer Agents) 22 (4), 731–746. 10.2174/1871520621666211013115500 34645380

[B50] Shannon P. Markiel A. Ozier O. Baliga N. S. Wang J. T. Ramage D. (2003). Cytoscape: a software environment for integrated models of biomolecular interaction networks. Genome Res. 13 (11), 2498–2504. 10.1101/gr.1239303 14597658 PMC403769

[B51] Sheng Q. Liu J. (2011). The therapeutic potential of targeting the EGFR family in epithelial ovarian cancer. Br. J. cancer 104 (8), 1241–1245. 10.1038/bjc.2011.62 21364581 PMC3078592

[B52] Tang Z. Kang B. Li C. Chen T. Zhang Z. (2019). GEPIA2: an enhanced web server for large-scale expression profiling and interactive analysis. Nucleic acids Res. 47 (W1), W556–W560. 10.1093/nar/gkz430 31114875 PMC6602440

[B53] Vellingiri B. Iyer M. Devi Subramaniam M. Jayaramayya K. Siama Z. Giridharan B. (2020). Understanding the role of the transcription factor Sp1 in ovarian cancer: from theory to practice. Int. J. Mol. Sci. 21 (3), 1153. 10.3390/ijms21031153 32050495 PMC7038193

[B54] Viveky N. Thorpe L. Alcorn J. Hadjistavropoulos T. Whiting S. (2015). Safety evaluation of flaxseed lignan supplementation in older adults residing in long-term care homes. JNHR-J Nurs. Home Res. 1, 84–88. 10.14283/jnhrs.2015.17

[B55] Walsh T. King M.-C. (2007). Ten genes for inherited breast cancer. Cancer Cell. 11 (2), 103–105. 10.1016/j.ccr.2007.01.010 17292821

[B56] Zoete V. Cuendet M. A. Grosdidier A. Michielin O. (2011). SwissParam: a fast force field generation tool for small organic molecules. J. Comput. Chem. 32 (11), 2359–2368. 10.1002/jcc.21816 21541964

