# Supplementary material for: From seeds to survival rates: investigating Linum usitatissimum’s potential against ovarian cancer through network pharmacology
Source: Front Pharmacol. 2023 Oct 30;14:1285258. doi: 10.3389/fphar.2023.1285258 (PMC10642394; doi:10.3389/fphar.2023.1285258)
Supplement: Supplementary file 1 [file Table2.DOCX]

**Table S1:** Comprehensive Analysis of Hub Genes: Identification and Characterization Based on Their Interaction with Specific Active Compounds from Pharmacological Screens

| **Genes** | **Compounds** |
| --- | --- |
| AKT1 | Apigenin |
| AKT1 | Vitamin E |
| AKT1 | Pantothenic acid |
| SRC | 5-Dehydro-avenasterol |
| SRC | Cholesterol |
| SRC | Campesterol |
| SRC | beta-Sitosterol |
| SRC | Stigmasterol |
| SRC | Apigenin |
| VEGFA | Daucosterol |
| MAPK3 | Isolariciresinol |
| MAPK3 | Cholesterol |
| MAPK3 | Campesterol |
| MAPK3 | beta-Sitosterol |
| MAPK3 | Stigmasterol |
| EGFR | Vitexin |
| EGFR | Daucosterol |
| EGFR | Apigenin |
| EGFR | Pantothenic acid |
| HSP90AA1 | Nicotinic acid |
| HSP90AA1 | Daucosterol |
| STAT3 | Daucosterol |
| JUN | Isolariciresinol |
| CASP3 | Pantothenic acid |
| ESR1 | Campesterol |
| ESR1 | beta-Sitosterol |
| ESR1 | Stigmasterol |
| ESR1 | Apigenin |
| ESR1 | Vitamin E |
| ESR1 | Isolariciresinol |
| ESR1 | 5-Dehydro-avenasterol |
| ESR1 | Cholesterol |

**Table S2:** Disease-Relevant Pathways: A Detailed Overview of Pathways in which the Identified Top Hub Genes Play a Predominant Role, Based on Functional Analysis

| **Genes** | **Pathways** |
| --- | --- |
| AKT1 | hsa05200:Pathways in cancer |
| AKT1 | hsa04020:Calcium signaling pathway |
| AKT1 | hsa05205:Proteoglycans in cancer |
| AKT1 | hsa05417:Lipid and atherosclerosis |
| AKT1 | hsa04931:Insulin resistance |
| AKT1 | hsa04010:MAPK signaling pathway |
| AKT1 | hsa01521:EGFR tyrosine kinase inhibitor resistance |
| AKT1 | hsa05207:Chemical carcinogenesis - receptor activation |
| AKT1 | hsa04750:Inflammatory mediator regulation of TRP channels |
| AKT1 | hsa04012:ErbB signaling pathway |
| AKT1 | hsa04080:Neuroactive ligand-receptor interaction |
| AKT1 | hsa05215:Prostate cancer |
| AKT1 | hsa04066:HIF-1 signaling pathway |
| AKT1 | hsa04024:cAMP signaling pathway |
| AKT1 | hsa04151:PI3K-Akt signaling pathway |
| AKT1 | hsa04722:Neurotrophin signaling pathway |
| AKT1 | hsa05161:Hepatitis B |
| AKT1 | hsa05135:Yersinia infection |
| SRC | hsa05167:Kaposi sarcoma-associated herpesvirus infection |
| VEGFA | hsa04020:Calcium signaling pathway |
| VEGFA | hsa05205:Proteoglycans in cancer |
| VEGFA | hsa04010:MAPK signaling pathway |
| VEGFA | hsa01521:EGFR tyrosine kinase inhibitor resistance |
| VEGFA | hsa05207:Chemical carcinogenesis - receptor activation |
| VEGFA | hsa04066:HIF-1 signaling pathway |
| VEGFA | hsa04151:PI3K-Akt signaling pathway |
| VEGFA | hsa05167:Kaposi sarcoma-associated herpesvirus infection |
| MAPK3 | hsa05200:Pathways in cancer |
| MAPK3 | hsa05205:Proteoglycans in cancer |
| EGFR | hsa05200:Pathways in cancer |
| EGFR | hsa04020:Calcium signaling pathway |
| EGFR | hsa04010:MAPK signaling pathway |
| EGFR | hsa01521:EGFR tyrosine kinase inhibitor resistance |
| EGFR | hsa05207:Chemical carcinogenesis - receptor activation |
| EGFR | hsa04012:ErbB signaling pathway |
| EGFR | hsa05215:Prostate cancer |
| EGFR | hsa04066:HIF-1 signaling pathway |
| EGFR | hsa04151:PI3K-Akt signaling pathway |
| EGFR | hsa01522:Endocrine resistance |
| HSP90AA1 | hsa05200:Pathways in cancer |
| HSP90AA1 | hsa05417:Lipid and atherosclerosis |
| HSP90AA1 | hsa05207:Chemical carcinogenesis - receptor activation |
| HSP90AA1 | hsa04151:PI3K-Akt signaling pathway |
| HSP90AA1 | hsa04659:Th17 cell differentiation |
| STAT3 | hsa05200:Pathways in cancer |
| STAT3 | hsa05205:Proteoglycans in cancer |
| STAT3 | hsa05417:Lipid and atherosclerosis |
| STAT3 | hsa01521:EGFR tyrosine kinase inhibitor resistance |
| STAT3 | hsa05207:Chemical carcinogenesis - receptor activation |
| STAT3 | hsa04066:HIF-1 signaling pathway |
| STAT3 | hsa05161:Hepatitis B |
| STAT3 | hsa04659:Th17 cell differentiation |
| JUN | hsa05200:Pathways in cancer |
| JUN | hsa05417:Lipid and atherosclerosis |
| JUN | hsa04010:MAPK signaling pathway |
| JUN | hsa05207:Chemical carcinogenesis - receptor activation |
| JUN | hsa04012:ErbB signaling pathway |
| JUN | hsa04024:cAMP signaling pathway |
| JUN | hsa04722:Neurotrophin signaling pathway |
| JUN | hsa05161:Hepatitis B |
| JUN | hsa05135:Yersinia infection |
| JUN | hsa04659:Th17 cell differentiation |
| JUN | hsa01522:Endocrine resistance |
| JUN | hsa05167:Kaposi sarcoma-associated herpesvirus infection |
| CASP3 | hsa05200:Pathways in cancer |
| CASP3 | hsa05205:Proteoglycans in cancer |
| CASP3 | hsa05417:Lipid and atherosclerosis |
| CASP3 | hsa04010:MAPK signaling pathway |
| CASP3 | hsa05161:Hepatitis B |
| CASP3 | hsa05167:Kaposi sarcoma-associated herpesvirus infection |
| CASP3 | hsa05163:Human cytomegalovirus infection |
| ESR1 | hsa05200:Pathways in cancer |
| ESR1 | hsa05205:Proteoglycans in cancer |
| ESR1 | hsa05207:Chemical carcinogenesis - receptor activation |
| ESR1 | hsa01522:Endocrine resistance |
